# Persistence and Effect of a Multistrain Starter Culture on Antioxidant and Rheological Properties of Novel Wheat Sourdoughs and Bread

**DOI:** 10.3390/foods9091258

**Published:** 2020-09-08

**Authors:** Rossana Sidari, Alessandra Martorana, Clotilde Zappia, Antonio Mincione, Angelo Maria Giuffrè

**Affiliations:** Department of AGRARIA, Mediterranea University of Reggio Calabria, loc. Feo di Vito, 89122 Reggio Calabria, Italy; alessandra.martorana@unirc.it (A.M.); clotilde.zappia@unirc.it (C.Z.); amincione@unirc.it (A.M.); amgiuffre@unirc.it (A.M.G.)

**Keywords:** lactic acid bacteria, yeasts, starter, sourdough, bread, phenolics, DPPH, Texture Profile Analysis

## Abstract

Food consumers make decisions primarily on the basis of a product’s nutritional, functional, and sensorial aspects. In this context, this study evaluated the persistence in sourdough of a multistrain starter culture from laboratory to bakery plant production and the effect of the starter on antioxidant and rheological properties of sourdoughs and derived bread. *Lactobacillus sanfranciscensis* B450, *Leuconostoc citreum* B435, and *Candida milleri* L999 were used as a multispecies starter culture to produce a sourdough subsequently used to modify two traditional sourdoughs to make novel bread with improved health and rheological properties. Both these novel bakery sourdoughs showed the persistence of *L. sanfranciscensis* B450 and *C. milleri* L999, and showed a significantly different lactic acid bacteria (LAB) concentration from the traditional sourdoughs. The novel sourdough PF7 M had a higher phenolic content (170% increase) and DPPH (8% increase) than the traditional bakery sourdough PF7 F. The novel sourdough PF9 M exhibited an improvement in textural parameters. Further research would be useful on the bioavailability of bio-active compounds to obtain bread with improved characteristics.

## 1. Introduction

Sourdough technology, based on the use of dough fermented by lactic acid bacteria (LAB) and yeasts which coexist and establish stable interactions, has attracted interest since sourdough positively affects the nutritional, textural, and sensorial characteristics of cereal-based products [1,2,3,4].

It is well known that phenols, bioactive compounds widely present in wine, olive oil, and plant-based foods [5,6,7], including cereals and their products [8,9,10], are antioxidants and help prevent cardiovascular diseases, chronic inflammation, cancer, and diabetes [11,12,13,14,15].

Microorganisms determine changes in chemical compounds present in the raw material [16,17,18,19] affecting also the bioactive compounds within cereal products [20,21], particularly in sourdough derived products rather than in yeast- or chemically-leavened bakery products [4,22]. Sourdough fermentation also affects the rheological properties of dough [23] as well as the textural and sensorial characteristics of bread [2,24,25,26,27,28,29]. In different geographical regions, research has been carried out aimed at improving the level of bioactive compounds and/or sensorial characteristics of bread, using starters or fortification of wheat flour or a combination of the two [30,31,32,33,34,35,36,37,38]. However, sourdough exhibits a wide biodiversity, and the present research aims to investigate autochthonous Calabrian sourdoughs and strains.

In the southern Italian region of Calabria, the production of wheat sourdough bread, usually called either *Pane di grano*, *Pane tradizionale*, or *Pane casereccio*, is well established in many cities and villages. The mother dough, prepared either using soft or durum wheat flour, is stored and continuously propagated using traditional procedures handed down from one generation to another.

The biodiversity of the different artisanal bakery productions is poorly studied [39,40], and there have been studies neither on the antioxidant and sensorial properties of Calabrian sourdoughs and bread, nor on the modification of existing artisanal preparations using starters and technological procedures with an expected impact on the bread’s characteristics.

The need for research is highlighted both by the lack of information about existing traditional products, and by the current consumer demand for food which is healthier and more functional [41] as well as appealing to the senses [42,43]. Research into traditional products would not only reveal the characteristics of traditional artisanal sourdoughs, but would also indicate a starting point for new formulations. Consumer demand for healthier bread justifies the creation of a multistrain starter able to outgrow the original microbiota of artisanal sourdoughs. For these reasons, autochthonous wheat-related Calabrian strains, selected based on useful technological properties, were chosen (see Material and Methods section for details) to make the starter culture used in this study.

The suitability of a starter is firstly determined by its stability over time, thus maintaining a certain reproducibility of a loaf’s characteristics, and secondly, by verifying its role and impact on the products. Therefore, the main aims of this work were to verify the persistence in sourdough of the multistrain starter culture—*Lactobacillus sanfranciscensis*, *Leuconostoc citreum*, and *Candida milleri*—throughout all stages of production (from laboratory to bakery plant) and to evaluate its role in modifying the content of polyphenols, the antioxidant capacity, and the textural properties of novel produced sourdoughs and bread.

There are two novel aspects of this work: firstly, it sheds light on artisanal sourdough biodiversity, and secondly, it combines artisanal knowledge of sourdough bread-making with the scientific basis for producing new bread.

## 2. Materials and Methods

Starter strains: Two strains of LAB, *L. sanfranciscensis* B450 and *L. citreum* B435, and a yeast strain, *C. milleri* L999, were used as multispecies starter culture to produce a sourdough—in this study named SD. These strains were previously isolated from artisanal sourdoughs and selected for their properties useful in bread-making [40]: the production of CO_2_, a fast acidifying activity, the production of exopolysaccharides, and the exhibition of proteolytic activity. The yeast *C. milleri* has a known stable association with *L. sanfranciscensis* due to maltose metabolism. In particular the strain L999 was chosen for its resistance to high salt concentration, tolerance to low pH, and growth in the presence of acetic acid.

Strains B450 and B435 were propagated overnight at 30 °C in Sour Dough Bacteria (SDB) broth [44] and in de Man–Rogosa–Sharpe (MRS) broth (VWR International s.r.l., Milan, Italy), respectively. Strain L999 was propagated overnight at 30 °C in Yeast Peptone Dextrose (YPD) Broth (Amresco, Milan, Italy).

Sourdough and bread production: Each overnight culture was harvested by centrifugation (5000 rpm for 10 min), washed once in 0.9% NaCl solution, resuspended to OD_600_ of 1.0 in the same solution, and used to prepare SD sourdough. Inoculated (SD) and noninoculated control (SDC) sourdoughs (500 g) were prepared in duplicate using durum wheat remilled semolina (Industria molitoria Minnini s.r.l, Italy) and sterile still mineral water with a dough yield (weight of the dough/weight of the flour × 100) of 170 that gave the best kneading performance. The raw ingredients for SD were inoculated with the prepared multispecies starter culture at 1% of the dough’s total volume. Both SD and SDC sourdoughs were kneaded by gloved hands in sterile stainless-steel trays, covered with plastic wrap, and incubated at 25 °C for 21 h to allow fermentation. Afterwards, sourdough propagations were carried out every two days and, after leavening, the sourdoughs were stored at 4 °C. After two months, SD and SDC sourdoughs were kneaded by a spiral kneading machine (CHEF Planetary mixer, Sigma s.r.l., Torbole Casaglia (BS), Italy) for 5 min and left to ferment for 4 h at 30 °C before baking 12 loaves × 500 g (6 for each type of sourdough) in an oven (BE-1 System Oven, Angelo Po Grandi Cucine s.p.a., Carpi (MO), Italy) at 200 °C for 15 min followed by a further 5 min at 180 °C.

**Figure 1:**
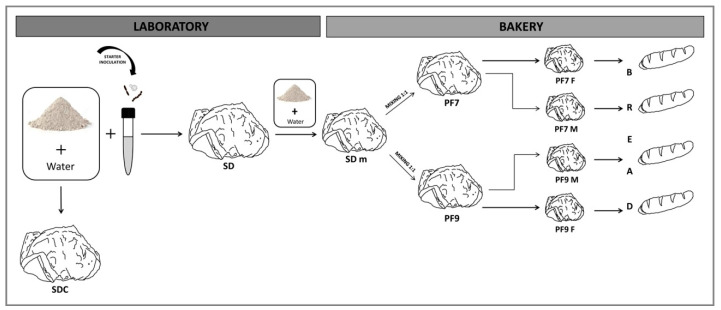
Experimental design.

In a bakery plant experiment, the SD sourdough propagated in laboratory (mother dough-SD m) was mixed both with artisanal Calabrian sourdoughs PF7 and PF9 [40], here used as mother doughs, in a ratio of 1:1 to produce novel sourdoughs named PF7 M (SD m + PF7) and PF9 M (SD m + PF9). The PF7 and the PF9 are made by two different bakeries using their traditional mother dough. In detail, PF7 M and PF9 M were composed of soft wheat flour type 00, 20% mother dough, 2% salt, and 60% tap water. The PF7 M and PF9 M sourdoughs were kneaded by a spiral kneading machine (Mecnosud s.r.l., Flumeri (AV), Italy) for 15 min and left to ferment for 4 h at 25 °C before baking at 250 °C for 30 min 16 loaves × 500 g for each type of sourdough. The same procedural scheme was followed for the bakery sourdoughs PF7 F and PF9 F to produce traditional bakery loaves (Figure 1).

Microbiological analysis: Plate counting was performed in triplicate for SD and SDC throughout 0–1140 h (2 months), for the SD m, PF7, PF9, PF7 F, PF9 F, for sourdoughs PF7 M (SD m + PF7), and PF9 M (SD m + PF9), and for PFC (baker’s yeast dough) as a control dough. After this, the sourdoughs were homogenized in 0.9% NaCl solution (1:10) and then diluted ten-fold. LAB were plated onto MRS agar and SDB agar supplemented with 100 mg/L cycloheximide (Oxoid, Milan, Italy), while yeasts were plated onto YPD agar (Amresco, Milan, Italy) supplemented with 100 mg/L chloramphenicol (Liofilchem Diagnostici, Roseto degli Abruzzi, Italy). Plates were incubated at 30 °C for 48 h anaerobically and aerobically for LAB and yeasts, respectively.

Monitoring of the added multistrain starter: Colonies from SD and SDC (0–21 h; 2 months), PF7 M and PF9 M (before baking) were randomly isolated based on their appearance and purified by restreaking on the above reported growth media. The presumptive LAB were tested for catalase and for Gram by the KOH method [45]. All the purified isolates were stored at −80 °C. To verify the persistence of the added strains to the experimental sourdoughs, each pure culture was subjected to DNA extraction by InstaGene Matrix (Bio-Rad Laboratories, Milan, Italy) according to the manufacturer’s instructions, and then analyzed for Random Amplified Polymorphic DNA (RAPD)—PCR technique. PCRs were performed in a MasterCycler Nexux GX2 (Eppendorf, Milan, Italy) using the primer M13 in a 25 µL reaction as reported by [46]. PCR amplicons were electrophoretically separated on 1.5% agarose gel using the GeneRuler 100 bp Plus (Thermo Fisher Scientific, Monza, Italy) as a ladder. The gels were stained with RealSafe Nucleic Acid Staining Solution (Real, Paterna, Valencia, Spain), checked under UV transillumination, and documented by the MicroDoc system (Cleaver Scientific, Warwickshire, UK).

The polymorphic profiles obtained from the pure cultures were compared with profiles of the axenic strains B450, B435, and L999 inoculated as starters.

PCR-Denaturant Gradient Gel Electrophoresis analysis: Sourdough SD and SDC were subjected to PCR-DGGE of a portion of the 16S and 26S rRNA [47]. LAB reference strains were: *Lactobacillus plantarum* subsp. *plantarum* LMG 06907^T^, *Lactobacillus pentosus* LMG 10755^T^, *Lactobacillus sanfranciscensis* LMG 16002^T^, *Lactobacillus brevis* LMG 07944^T^, *Lactobacillus buchneri* LMG 06892^T^, *Lactobacillus fructivorans* LMG 09201^T^, *Lactobacillus reuterii* LMG 09213^T^, *Pediococcus pentosaceus* LMG 11488^T^, *Lactobacillus pontis* LMG 14187^T^, *Lactococcus lactis* subsp. *lactis* LMG 06890^T^, and *Pediococcus acidilactici* LMG 11384^T^ together with *L. sanfranciscensis* B450 and *L. citreum* B435, previously isolated [40]. Yeasts reference strains were: *C. milleri* CBS 6897^T^, *Kluyveromyces marxianus* CBS 834^T^, *Saccharomyces cerevisiae* CBS 1171^T^, *Wickerhamomyces anomalus* CBS 5759^T^ together with *C. milleri* L999, previously isolated [40]. PCR products were analyzed by the D-code apparatus (Bio-Rad Laboratories) loading them onto 8% (*w*/*v*) polyacrylamide gels (acrylamide/bis-acrylamide, 37.5:1) in 1× TAE buffer containing 30% to 60% urea-formamide linear denaturating gradient increasing in the direction of electrophoresis. The electrophoresis parameters were 100 V with a running temperature of 60 °C for 7 h. After staining, DNA from bands of interest was reamplified; then, the PCR products were purified by Illustra™ GFX™ PCR DNA and Gel Band Purification Kit (GE Healthcare, Freiburg, Germany) and sequenced (Eurofins Genomics, Ebersberg, Germany). The sequences obtained were compared with those available at NCBI by the Blast search tool [48] and submitted to GenBank for accession numbers (see Results section).

Chemical and rheological analyses: These analyses were carried out on sourdoughs and/or breads made in the laboratory and at the bakery. Total phenolic content, DPPH, and texture parameters were also performed on ten (PF1–PF10) artisanal Calabrian sourdoughs. The PFC dough produced with baker’s yeast supplied by a local bakery was used as a control. After baking, the loaves were left to cool and then analyzed, sampling both crust and crumb.

In detail, pH and total titratable acidity (TTA) were carried out in triplicate on SD; SDC sourdoughs (0–2–4–6–8–21 h); PFC (laboratory experiments); and on SD m, PF7, PF9, PF7 F, PF9 F, PF7 M, and PF9 M sourdoughs (bakery experiments). The pH was determined by a spin electrode pH meter (HI99161, Hanna Instruments, Ronchi di Villafranca Padovana, Italy), and the total titratable acidity (TTA) was determined by titration using 0.1 N NaOH on 10 g of each sample and expressed as mL of NaOH.

The organic acids were detected for SD; SDC sourdoughs/breads (laboratory experiments: 0-8-21 h and 2 months); PFC dough; the mother doughs for the bakery experiment (SD m, PF7, and PF9); PF7 F, PF9 F, PF7 M, and PF9 M sourdoughs/breads (bakery experiments). The organic acid extraction was carried out in triplicate according to Martorana et al. [40]. In brief, sourdough homogenates were centrifuged at 5000× *g* for 15 min, and the supernatant was filtered with 0.45 μm PTFE filter (Supelco Sigma-Aldrich, Milan, Italy). Then, the obtained water-/salt-soluble extract was analyzed by HPLC equipped with an Acclaim OA 5 μm (4 × 250 mm) at 30 °C and with the UV detector operating at 210 nm, with a flow rate of 0.6 mL/min The isocratic mobile phase was 100 mM Na_2_SO_4_ acidified with methanesulfonic acid to a pH of 2.65. External standard method was used to quantify the compounds detected. Data were expressed as mg/g. The quotient of fermentation (QF) was calculated as the molar ratio between d, l-lactic and acetic acids.

To determine total phenolic content and antioxidant activity, five grams of each sourdough/bread was added to 50 mL of 80% methanol, mixed for 30 min, and centrifuged at 6000× *g* for 20 min. The obtained extracts were analyzed for phenolic content by the Folin–Ciocalteu method [49] and by the 2,2-diphenyl-1-picrylhydrazyl radical (DPPH) free radical scavenging method [50].

Rheological analyses were performed on sourdoughs and breads by a TA-XT Plus Texture Analyzer (Stable Micro Systems Ltd., Godalming, UK). Data acquisition and curve integration were carried out by Exponent software 6.1.4.0 (Stable Micro Systems Ltd., Godalming, UK).

The PF1-PF10 sourdoughs; mature SD and SDC sourdoughs; PFC; the mother sourdoughs SD m, PF7, PF9; the traditional fermented sourdoughs PF7 F and PF9 F; and the novel sourdoughs PF7 M and PF9 M were subjected to the Stickiness, Penetration, Warburtons, and Kieffer tests.

The Stickiness test was performed using a Chen and Hoseney probe (A/DSC) (Stable Micro Systems Ltd., Godalming, UK) on 20 g of sample. The probe in contact with the sample measured the forces of insertion and withdrawal from the dough. To evaluate this attribute, the following parameters were used: pretest speed: 0.50 mm/s; test speed: 0.50 mm/s; post-test speed: 10.00 mm/s; distance: 4.0 mm; trigger force: 5.0 g; data acquisition rate: 400 pps. For each sample, ten replicates were carried out. In order to assess the hardness of the dough, the Penetration test was performed on 110 g of sample using a 6 mm cylindrical probe (P/6) (Stable Micro Systems Ltd., Godalming, UK). The set parameters were: pretest speed: 2.00 mm/s; test speed: 3.00 mm/s; post-test speed: 10.00 mm/s; distance: 20.0 mm; trigger force: 5.0 g; data acquisition rate: 200 pps. For each sample, six replicates were carried out. The Warburtons test was performed using the Warburtons dough stickiness system (A/WDS500) (Stable Micro Systems Ltd., Godalming, UK) on 500 g of sample. The sample, placed into a box, is slightly compressed by a retaining plate with a slot in the middle. Then, a blade is driven through the slot to obtain indications of its consistency. The test was carried out applying the following parameters: pretest speed: 5.00 mm/s; test speed: 2.00 mm/s; post-test speed: 10.00 mm/s; distance: 40.0 mm; trigger force: 10.0 g; data acquisition rate: 500 pps. For each sample, five replicates were carried out. The Kieffer test was performed using a Kieffer probe (A/KIE) (Stable Micro Systems Ltd., Godalming, UK) on 50 g of sample. In this test, the dough is stretched by the probe. The following parameters were used: pretest speed: 2.00 mm/s; test speed: 3.30 mm/s; post-test speed: 10.00 mm/s; distance: 75.0 mm; trigger force: 5.0 g; data acquisition rate: 400 pps. For each sample, ten replicates were carried out.

Concerning the bread, the PF7 F, PF9 F, PF7 M, and PF9 M breads were analyzed for the Penetration test, Cut slice test, and Texture Profile Analysis (TPA). Three replicate measurements were made on bread samples.

A single whole loaf was used in the Penetration test to assess its hardness and fracturability. The test was performed using a 3 mm cylindrical probe (P/3) (Stable Micro Systems Ltd., Godalming, UK) that was driven into the bread with the following parameters: pretest speed: 1.00 mm/s; test speed: 0.50 mm/s; post-test speed: 10.00 mm/s; distance: 10.0 mm; trigger force: 5.0 g; data acquisition rate: 400 pps. For each sample, three replicates were carried out. Moreover, the hardness was evaluated on slices of bread. In detail, 2 cm thick slices were analyzed by the Cut Slice test using a Blade Set with Knife probe (HDP/BSK) (Stable Micro Systems Ltd., Godalming, UK). The set parameters were: pretest speed: 1.50 mm/s; test speed: 2.00 mm/s; post-test speed: 10.00 mm/s; distance: 30.0 mm; trigger force: 25.0 g; data acquisition rate: 400 pps. For each sample, three replicates were carried out.

The TPA test was performed using a 100 mm compression platen (P/100) probe (Stable Micro Systems Ltd., Godalming, UK) on a whole loaf sample with the following parameters: pretest speed: 1.00 mm/s; test speed: 5.00 mm/s; post-test speed: 5.00 mm/s; distance: 20.0 mm; trigger force: 5.0 g; data acquisition rate: 400 pps. For each sample, three replicates were carried out. From test results, the chewiness, springiness, and resilience parameters were taken into consideration.

Statistical analyses: Excel 2010 software (Microsoft, Milan, Italy) was used to calculate the means and standard deviations on three replicates. The means were analyzed by one-way ANOVA and a Tukey’s test, at 5% probability, using the SPSS 17.0 software (SPSS Inc., Chicago, IL, USA).

## 3. Results

### 3.1. Microbial Count

LAB and yeast load reflects the majority population in sourdoughs; their count and the ratio between them, with LAB dominating the yeasts, depict the quality of a sourdough and allow the progress of fermentation to be tracked. Moreover, high LAB concentration prevents undesired bacteria such as *Enterobacteriaceae* taking advantage and proliferating.

**Figure 2:**
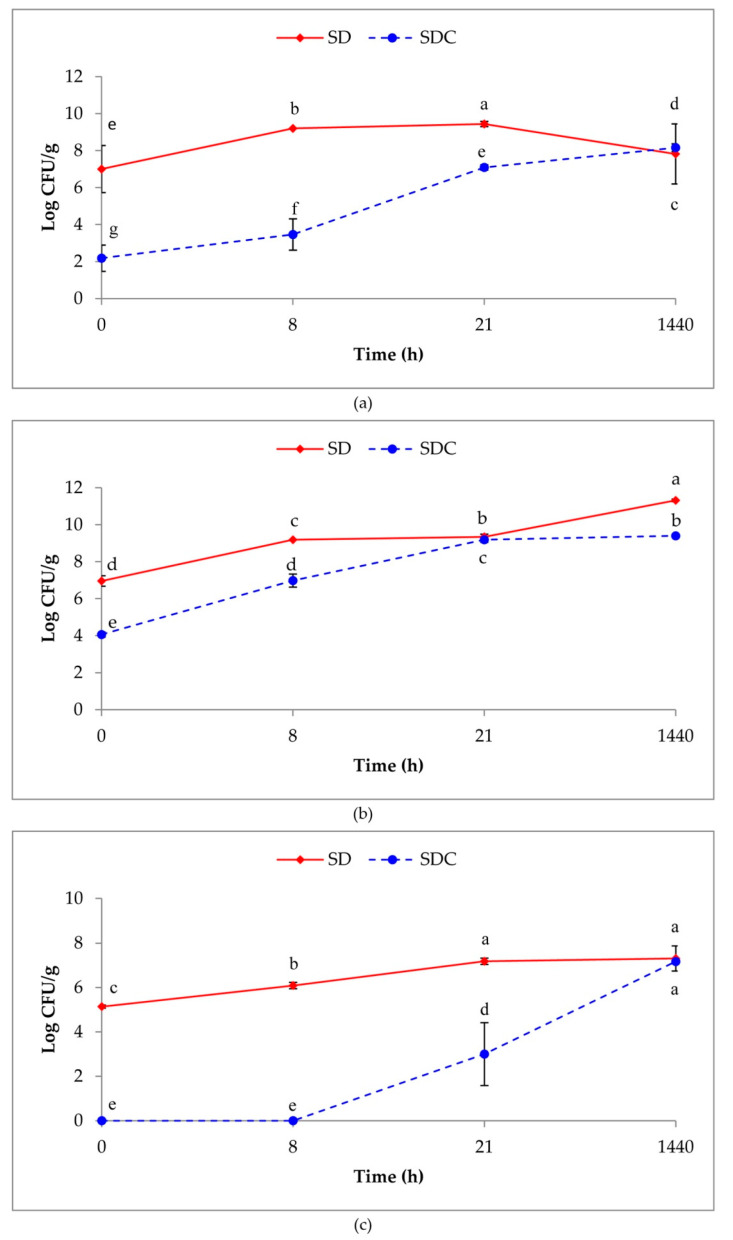
Microbial loads of SD (inoculated with starter) and SDC (uninoculated) sourdoughs. Lactic acid bacteria (LAB) detected in de Man–Rogosa–Sharpe (MRS) (**a**) and Sour Dough Bacteria (SDB) agar (**b**); yeasts detected in Yeast Peptone Dextrose (YPD) agar (**c**). Loads in different media were analyzed separately and different letters indicate significant differences according to the Tukey’s test (*p* ≤ 0.001).

Concerning the laboratory experiment, just after the sourdough preparation (0 h), the LAB load was higher in SD (10^7^ CFU/g) than in SDC (10^2^–10^4^ CFU/g in MRS and SDB, respectively) (Figure 2). At the start of fermentation, yeasts were detected only in SD. These results indicate that LAB and yeasts level in SD were due to the multistrain starter addition. Moreover, 6.7% of the bacterial colonies isolated from SDC were different from LAB since they were catalase positive and Gram negative. Until 21 h of fermentation, in SD, the LAB load increased by about two orders of magnitude in both media, whereas in SDC, the LAB load gradually increased with time, remaining always lower than the SD values. After 2 months, the LAB detection in MRS medium registered a decrease of about 2 Log for SD and a slight increase in SDC. The opposite was observed in SDB medium where the LAB load for SD reached the highest values. The yeast load was in the range 10^5^–10^7^ CFU/g in SD, while in SDC, the yeasts were below the detection limit until 8 h; then, it gradually increased up to 10^7^ CFU/g. The yeast and LAB load detected in PFC was 10^9^ and 10^4^ CFU/g.

**Figure 3:**
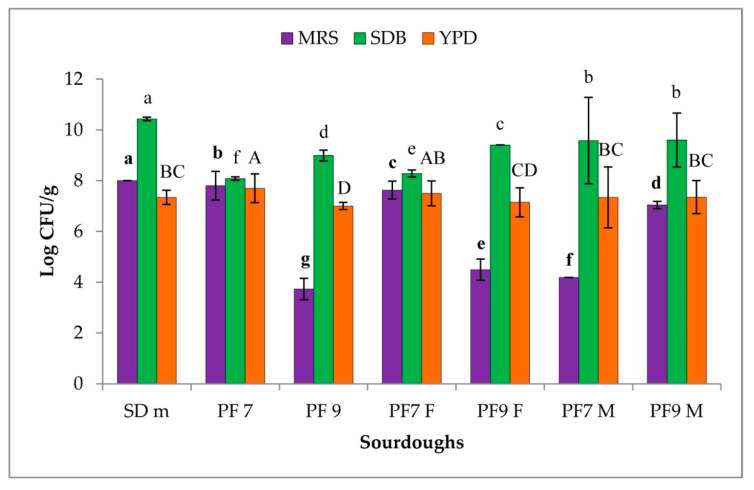
Microbial loads at bakery experiment. Mean values with different letters were significantly different according to the Tukey’s test (*p* ≤ 0.001). Media were analyzed separately and distinguished with bold (MRS), small (SDB), and capital letters (YPD), respectively.

Figure 3 reports results of the trials carried out at the bakery plant. Comparing the mother doughs, higher LAB loads were detected in SD m than in the others. For all of the samples, higher levels of LAB were registered on SDB rather than MRS. Novel sourdoughs PF7 M and PF9 M were characterized by lower LAB loads in MRS than in SDB, more marked in PF7 M. Additionally, for the traditional bakery sourdoughs, the highest LAB load was detected in SDB medium. For all the trials the yeast load was similar with a value of 10^7^ Log CFU/g. Novel sourdoughs were significantly different from the respective traditional ones regarding the LAB concentration.

### 3.2. Persistence of the MultiStrain Starter

To follow the persistence of the multistrain starter during the SD propagation and in the novel bakery sourdoughs, the RAPD profiles of the strains isolated from each sourdough were compared to those of the three pure cultures forming the starter used to make sourdough.

The LAB population of the SD sourdough from 0 to 21 h of fermentation was dominated by both *L. citreum* B435 and *L. sanfranciscensis* B450. At the start of fermentation, the former slightly dominated over the latter; subsequently, their presence was comparable and at 21 h, the *L. sanfranciscensis* B450 became dominant. At 2 months, only *L. sanfranciscensis* B450 was detected. Regarding the yeast, the *C. milleri* L999 dominated throughout the fermentation and propagation time of SD. The profiles of the multistrain starter added were not associated with any profiles of the SDC isolates.

Both novel bakery sourdoughs PF7 M and PF9 M showed the persistence of *L. sanfranciscensis* B450 and *C. milleri* L999. This finding was consistent with the starter composition of SD used as the mother dough in bakery trials. For both sourdoughs, LAB and yeast profiles different from those of the starters were detected; therefore, taking into account the profile of the total number of isolates, the PF7 M was characterized by 50% of *L. sanfranciscensis* B450 and 40% of *C. milleri* L999, while the PF9 M by 50% of *L. sanfranciscensis* B450 and 60% of *C. milleri* L999. Moreover, the PF9 M LAB profiles, different from the profile associated with *L. sanfranciscensis* B450, showed more biodiversity compared those detected for PF7 M (data not shown).

### 3.3. Microbial Community Fingerprinting by PCR-DGGE

DGGE analysis of 16S and 26S rRNA allowed us to investigate bacteria and yeast communities of the inoculated SD and the control SDC sourdoughs. The size of the PCR amplicons obtained using the primers HDA6-f/L1395-r for the 16S rRNA gene of the bacteria was 218 bp, while the size of the PCR amplicons obtained using the primers LIEV-f/LIEV-r for the 26S rRNA gene of the yeasts was 214 bp. The SD and SDC bacteria population exhibited different profiles. The tentative species assignment by comparison with the profile of the reference strains was confirmed by band sequencing. The majority of the bands corresponded to LAB, with the exception of a few which had a 95% identity with a portion of the mitochondrial 18S rDNA of *Triticum aestivum* and some bands of SDC sourdoughs throughout 0–21 h of fermentation, which corresponded to other bacterial groups.

Throughout 0–21 h of fermentation no differences in the SD bacterial population were observed; it was composed of *L. citreum* (99% of similarity; accession number: MT457655) and *L. sanfranciscensis* (99% of similarity; accession number: MT457656). On the contrary, at 2 months, only *L. sanfranciscensis* (99% of similarity; accession number: MT457657) was detected. As regards the yeast SD population at 0 h, two bands were detected and they corresponded to *Wickerhamomyces anomalus* and *Candida humilis*; subsequently, all the bands corresponded to *C. humilis* until 2 months. The accession numbers of the bands sequenced and deposited to GenBank are: MT460390 with 100% of similarity for the band associated with *W. anomalus* and MT460390 with 100% of similarity for the band associated with *C. humilis*.

Concerning the bacteria SDC population, all the bands at 0–21 h of fermentation were associated to non-LAB; in detail, bands corresponded to *Cronobacter turicensis* (94% of similarity; accession number: MT457658), *Pantoea agglomerans* (99% of similarity; accession number: MT457659). At 2 months, the band corresponded to *L. sanfranciscensis* (MT457660). For the SDC yeast profiles, they were associated with *W. anomalus* (100% of similarity; accession number: MT460392).

### 3.4. Chemical Characterization of Sourdoughs and Breads

#### 3.4.1. pH, TTA, and Organic Acids

The pH and TTA parameters allow the sourdough development to be followed and they depend on LAB and yeast growth and metabolism.

**Figure 4:**
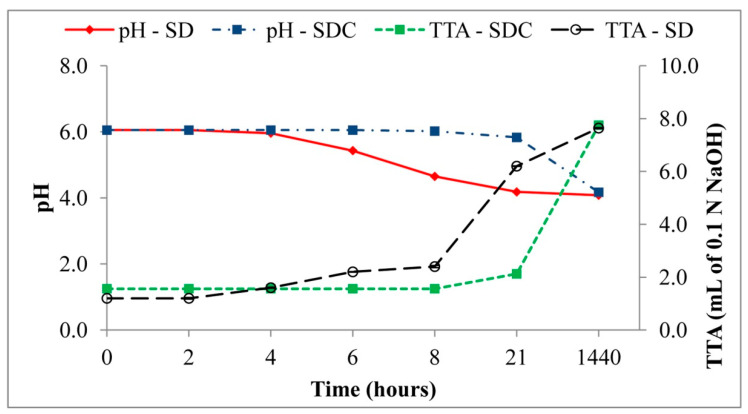
pH and total titratable acidity time course of SD (inoculated with starter) and SDC (uninoculated) sourdoughs produced in laboratory.

Figure 4 reports the pH and TTA kinetics of SD and SDC sourdoughs throughout the 21 h of fermentation and after 2 months of propagation. At the start, both sourdoughs had a pH value of 6.05; then, their pH evolved differently. The inoculated sourdough exhibited a pH decrease starting from the third hour reaching at 21 h the final value of 4.18. On the contrary, the SDC maintained the initial pH till 8 h still showing a high pH at 21 h. After 2 months of propagation, the pH was similar for SD and SDC (4.08 and 4.17, respectively). The PFC, baker’s yeast dough, had a pH of 5.18 ± 0.01. The TTA values were linearly correlated to the pH and ranged from 1.00 to 7.64 for PFC and SD at 2 months, respectively. Both the pH and TTA values were distributed in seven homogeneous groups (a homogeneous group defined as a group of means within which there are no statistically significant differences), and the differences between groups were statistically significant (*p* ≤ 0.001).

Among the mother doughs, the SD m had the lowest pH and the highest TTA followed by PF9 and PF7. Before baking, the PF9 M exhibited the minimum value of pH, whereas the PF7 F exhibited the maximum. Additionally, for this experiment, the TTA was linearly correlated to the pH (Table 1).

For the laboratory experiment, the initial concentration (0 h) of lactic acid, expressed as mg/g, was 0.14 ± 0.00 and 0.11 ± 0.00 for SD and SDC, respectively, while the acetic acid was 0.05 ± 0.01 and 0.06 ± 0.02 for SD and SDC, respectively. As the fermentation proceeded, a rapid increase in lactic acid for SD, which also maintained a low acetic acid concentration, was observed; this was in contrast with the small increase in lactic acid for SDC till 21 h (0.13 ± 0.02 mg/g), which also exhibited a higher acetic acid level than SD. The PFC was characterized by low levels of lactic (0.12 ± 0.01 mg/g) and acetic acid (0.14 ± 0.06 mg/g).

Lactic and acetic acids are the most important organic acids in sourdoughs produced by LAB and yeasts. Their concentration affects structure, sensorial characteristics, and shelf-life of the products [51].

Table 2 shows the organic acid concentration and the QF of sourdoughs and bread produced in the laboratory (2 months) and at the bakery. The inoculated sourdough exhibited higher and lower levels of lactic and acetic acids, respectively, than SDC. As regards the bakery experiment, the mother dough SD m was characterized by higher values of lactic and acetic acids (4.60 and 0.87, respectively) than the mother doughs PF7 (0.34 and 0.42) and PF9 (3.64 and 0.42). Compared to the bakery traditional sourdoughs, the PF7 M showed an increase in lactic acid concentration, while the PF9 M had an opposite behavior. Both the modified sourdoughs had lower acetic acid values than the traditional ones. In general, the bread was characterized by lower organic acid concentration than the respective sourdoughs. The PF9 bread had more lactic acid than that using PF7, and the modified bread more than the traditional bakery bread. For both breads, the concentration of acetic acid decreased in the modified loaves. The different concentrations of these two organic acids had an impact on the aroma profile of the sourdough products that is better indicated by the QF. The sourdough QF ranged from 0.64 to 4.70 (see Discussion section).

#### 3.4.2. Total Phenols and DPPH Assay

Total phenol and antioxidant activity detection gives information on the possible health implication of bread consumption.

Among the ten Calabrian sourdoughs, the lowest phenolic content, expressed as mg of gallic acid/100 g of sourdough, was found in PF2 (97.64), whereas the highest was found in PF10 (195.62). The highest values were detected for sourdoughs made using durum wheat (PF1, PF3, PF4, and PF10) compared to the majority made with soft wheat (Table 3). The control dough exhibited, as expected, the lowest content of total phenols (71.61). The antioxidant activity (expressed as a percentage of inhibition) ranged from 14.14 (PF9) to 35.82 (PF10) (Table S1).

Throughout the propagation of SD and SDC sourdoughs, the phenolic content gradually increased reaching the highest values at two months. Overall, this trend was observed also for the DPPH scavenging activity until 21 h; then, the % DPPH inhibition decreased in both sourdoughs with the highest scavenging activity detected for SD (Table 3). The values of total phenols at two months were comparable to those reported for the Calabrian sourdoughs made with durum wheat (Table S1).

The SD bread is characterized by a higher phenolic content and DPPH inhibition than the SDC bread. Specifically, both breads exhibited an increase of DPPH inhibition (113% and 135% for SD and SDC, respectively) and a decrease in phenolic content (55% and 60% for SD and SDC, respectively) compared to their respective two-month sourdoughs (Table 3).

As regards the mother doughs used in the bakery trial, the DPPH inhibition ranged from 14.14% (PF9) to 22.93% (SD m), and the total phenolic content from 109.65 (PF7) to 158.97 mg/100 g (SD m). In Table 4, the phenolic content and DPPH inhibition of sourdoughs and breads made at the bakery, are presented. The PF9 F sourdough exhibited higher antioxidant activity than the PF7 F. Comparing traditional and novel sourdoughs, the novel sourdough PF7 M had a higher phenolic content (170% increase) and DPPH inhibition (8% increase) than the traditional bakery sourdough PF7 F, while the opposite was detected for the PF9 M that showed a decrease for both parameters (14% for total phenols and 33% for DPPH inhibition) when compared to the PF9 F.

All the breads produced at the bakery showed higher DPPH inhibition and lower phenolic content than the respective sourdoughs. In detail, the DPPH increase was 156% and 74% for PF7 F and PF9 F breads, respectively; while, for PF7 M and PF9 M it was 142% and 105%, respectively. The PF7 M bread exhibited an increase both of DPPH inhibition (2%) and phenolic content (43%) compared to the traditional PF7 F bread. On the contrary, the PF9 M bread showed a decrease (22%) both in DPPH inhibition and phenolic content when compared to the traditional bread PF9 F.

In some cases, sourdoughs with a lower phenolic content exhibited similar or higher DPPH inhibition than sourdoughs with a higher phenolic content.

#### 3.4.3. Rheological Characterization of Sourdoughs and Bread

The rheological parameters, such as stickiness [52,53], firmness, and extensibility [54], are commonly used to assess the characteristics of a dough. TPA is widely applied to determine food texture, which is one of the most studied attributes at both processing and consumption levels [55,56,57,58]. Stickiness affects dough processing or mixing; the Stickiness test helps to investigate this dough attribute related to overmixing, addition of excess water, overactivity of proteolytic enzymes, and difference in wheat varieties and composition [59]. The knowledge of rheological parameters such as dough firmness (kg), work of compression (kg·s), and adhesiveness (kg)—obtained by the Warburtons test—gives insight on dough consistency useful for minimizing processing problems for bakeries when dough is subjected to shear, such as in dividing, molding, or reshaping of dough pieces [60]. The main parameters obtained from the Kieffer test, resistance to extensibility (g) and extensibility (mm), are useful for monitoring and controlling the production process [61]. As regards the TPA, we focused on the main parameters considered useful in bread acceptance, such as chewiness, intended as the energy required to disintegrate a semisolid food until it is ready to swallow; springiness, as the rate at which a deformed sample reforms; and resilience, as the measurement of how a sample recovers from deformation in relation to speed and forces derived [62].

Table S2 reports rheological results from the ten artisanal Calabrian sourdoughs and the PFC control sourdough. In the Stickness test, the sourdough PF4 sample showed the higher values with 261.76 g for Stickiness, 15.04 g·s for Work of adhesion, and 3.76 mm for Dough strength/Cohesiveness; in the Penetration test, the highest value was shown by sourdough PF8 with a Hardness value of 281.76 g. Warburtons and Kieffer tests were not performed for all sourdoughs due to problems in sample handling. In fact, Warburtons data were not available for the PF2, PF4, and PF6 sourdoughs, while Kieffer test data were not obtained from the PF1 and PF4 sourdoughs. Warburtons Firmness highest value was obtained from the PF7 sample with 8.62 kg, while the PF7 sample scored the highest Work of compression value with 52.09 kg·s. Adhesiveness data gave, on the other hand, homogeneous results for all samples. For the Kieffer test, the PF9 sourdough scored the highest Resistance to extensibility value (44.84 g) and Extensibility value (16.76 g).

Table 5 and Table 6 report rheological parameters of the sourdoughs made in the laboratory and at the bakery, respectively. Concerning the rheological behavior of doughs tested, the SD and SDC sourdoughs reported higher values for the Stickness test (111.82 and 114.40 g, respectively), Work of Adhesion (19.22 and 16.82 g·s, respectively) and Dough strength/Cohesiveness (6.37 and 6.31 mm, respectively) compared to most bakery sourdoughs, with the exception of sourdough PF9 M, which showed comparable Stickiness value (105.49 g). On the other hand, in the Penetration test, the SD and SDC sourdoughs reported lower hardness values (46.37 and 42.06 g, respectively) compared to all bakery sourdoughs, with the exception of the sourdough PF7 F, which reported a value of 62.73 g. Additionally, in the Warburtons test, the behavior of the SD and SDC laboratory sourdoughs turned out to be different from the bakery sourdoughs, showing Firmness values of 0.59 and 0.47 kg, respectively, when compared with the 1.38 kg value recorded by the mother sourdough PF9 + SD m. Similar behavior occurred for both remaining parameters of the Warburtons test (Work of Compression and Adhesiveness peak). Finally, results from the Kieffer test showed that laboratory sourdoughs had lower Resistance to extensibility (with 6.48 and 5.22 g, respectively) and Extensibility values (with 0.23 and 0.19 mm, respectively) in comparison to all bakery sourdoughs.

**Figure 5:**
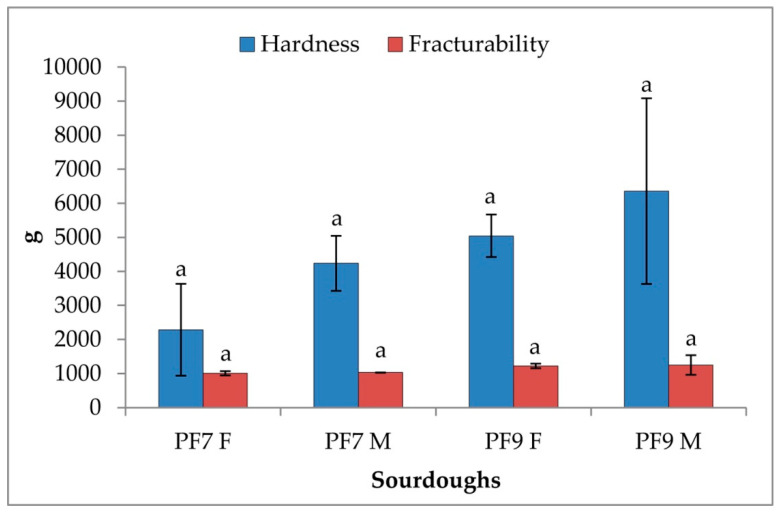
Penetration test of breads from traditional bakery and novel sourdough made at the bakery plant. Letters above the error bars refer to the statistical analysis.

The Penetration test performance of breads made from traditional bakery and novel sourdoughs is reported in Figure 5. Both novel sourdough breads exhibited a higher hardness compared to the traditional ones even if each sample did not give significantly different results; especially fracturability values were mostly uniform.

**Figure 6:**
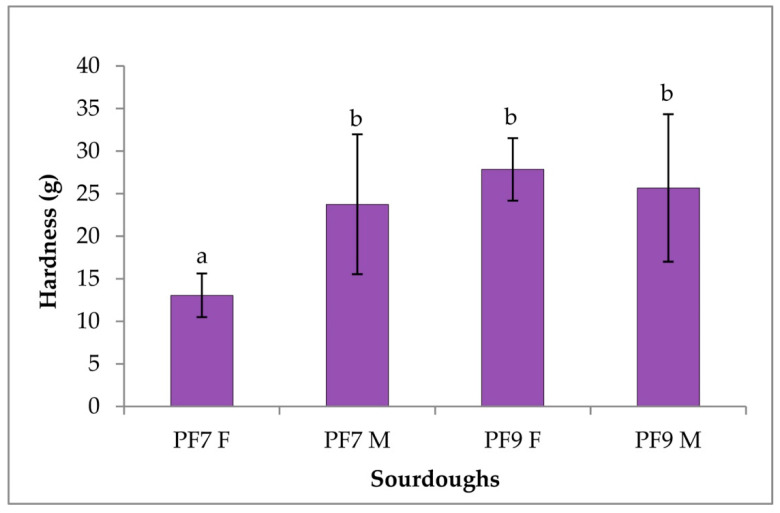
Cut slice test of the breads from traditional bakery and novel sourdoughs made at the bakery plant. Letters above the error bars refer to the statistical analysis.

Hardness assessed by bread cut slice tests on samples made from the traditional bakery’s and novel sourdoughs is reported in Figure 6. The addition of the multistrain starter mother sourdough determined a significant increase in hardness for the PF7 M bread compared to the traditional PF7 F, while the slight differences among PF9 F and PF9 M were not statically significant. The PF7 F sample result was statistically different from those of the other samples, which in turn were not statistically different.

**Figure 7:**
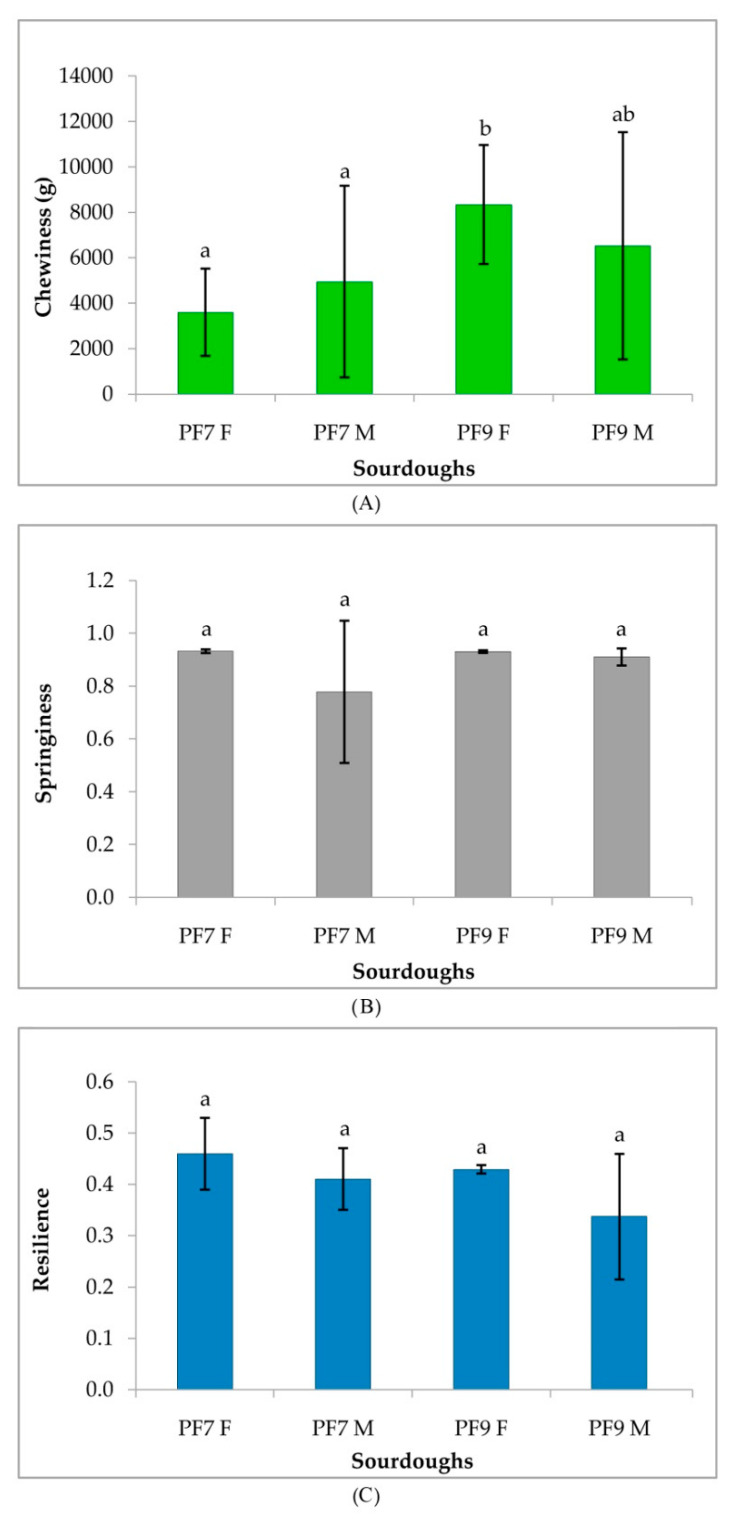
Texture Profile Analysis: (**A**) Chewiness, (**B**) Springiness, and (**C**) Resilience of breads made from traditional bakery and novel sourdoughs made at the bakery plant. Letters above the error bars refer to the statistical analysis.

Figure 7 reports the TPA results of breads made from traditional bakery and novel sourdoughs. Additionally, for the TPA parameters, in particular Chewiness and Springiness, the effect of the multistrain starter mother sourdough was different for the two novel formulated breads. From the three TPA parameters considered (Chewiness, Springiness, and Resilience), only chewiness data showed some significant differences, notably in bread samples from PF9 sourdough that behaved differently from PF7 and PF7 M samples. On the other hand, the PF9 M sample was found to be ascribable to both statistical groups.

## 4. Discussion

In this study, a multistrain starter culture was developed and used to manufacture novel wheat sourdoughs and breads exploiting its effect on antioxidant and rheological properties.

The results regarding microbial loads are consistent with those reported for traditional and experimental sourdoughs [30,35,37,40], and the pH and TTA evolution is comparable as well [40,51].

The concentration of lactic and acetic acids in our sourdough samples was similar to values reported by Settanni et al. [51]. To present an interpretation of the role of these organic acids, the QF is commonly reported [35,63,64]. The sourdough made in the laboratory and the novel sourdoughs can positively influence the aroma profile and the structure of the final products, having a QF in the range 1.5–4 that is considered positive in sourdough bread-making [65].

Different studies have reported the stability of the microbial population during continuous propagation of sourdough [30,35,66,67]. The mature sourdough SD made in the laboratory and the novel sourdoughs made at the bakery were dominated by *L. sanfranciscensis* and *C. milleri* confirming the key role of this LAB [68] and its strict association with *C. milleri* [63,69].

The PCR-DGGE analysis of the experimental sourdoughs revealed their microbial ecology throughout the propagation time. The size of the bands is in accordance with Gatto et al. [47]. The bands identified as *T. aestivum* and *C. turicensis* with a sequence identity lower than 97%, were poorly related to those deposited in the GenBank database. Nevertheless, the detection of *T. aestivum* is consistent with the primers used that also amplify plant material as reported by Gatto et al. [47]. Concerning the detection of *C. turicensis*, various authors have previously reported its presence in flour, cereal-based products, and bread [70,71,72,73]. Moreover, DGGE band sequencing demonstrated the presence of members of *Enterobacteriaceae* in the naturally fermented sourdough. Other authors have detected enterobacteria in sourdoughs [47,74]. Some members of enterobacteria such as *Enterobacter cowanii* and *P. agglomerans* have been isolated from nonsterilized flour [75]; therefore, it is highly probable that they derived from the flour used to prepare the sourdoughs SD and SDC. It is interesting to highlight the role of the multistrain starter added in SD in outgrowing the above reported harmful bacteria that instead dominated the SDC bacteria population up to 2 months.

The phenolic content is usually related to different factors other than the microbial composition. Although cereal grains are a good source of bioactive compounds, their level and activity are affected by cereal species and varieties, the nature of the antioxidants, the milling process producing flours with different degrees of refining, and food processing [1,76,77,78,79,80,81,82,83,84,85]. In detail, durum wheat has higher phenol and antioxidant activity than soft wheat [76,86]; similarly, whole flour and bread more than the refined [87,88,89,90], since phenolic compounds are mostly present in the outer layers of the grain that are usually removed by the milling industries [91].

Consistent with the above reported literature, lower values characterize the majority of PFs (see Table S1) made with soft wheat and the PFC (lowest value), which is, in addition, made with baker’s yeast. The phenolic content increase observed in the sourdough made in the laboratory (see Table 4) could be due to the progress of lactic acid fermentation that determines a pH decrease. The acidification, in fact, can enhance the phenol extractability, and LAB can determine the hydrolysis of complex phenolic forms shaping the phenolic profile of a matrix and its radical scavenging activity [1,20,92,93]. The higher values of SD compared to SDC could be attributable to the lower pH and higher LAB loads than the SDC values. These results are in agreement with the reported correlation between the antioxidant properties and the level of inoculum and pH value [94].

The difference in DPPH inhibition capacity observed between the novel sourdoughs could be due to the different LAB biodiversity revealed by the RAPD profiles, higher in PF9 M than in PF7 M; in fact, different microbial fermentation could lead to a variable effect due to the activation of inactive compounds but also deactivation of bioactive compounds [94].

Correlating phenolic content and antioxidant activity, our results could be attributable to the type of phenolics present in fermented doughs that possess different antioxidant activity [77]; therefore, the phenols associated with sourdoughs exhibiting lower phenolic content exert the same or higher antioxidant activity per mass unit compared to others. Similar results have been reported for barley and oat grains [95,96].

To the best of our knowledge, no studies have investigated phenols and antioxidant properties of artisanal wheat sourdoughs. The availability of various methods of detection and the different ways to express results do not make it easy to directly compare our results with those of others in the same field. However, our results are consistent with the trend reported by other authors, demonstrating that selected starters for bread-making increase the content of phenolic compounds, and the effect on antioxidant activity is not always positively correlated with the content of phenols [21,97,98,99].

Our results on breads are consistent with findings of several authors who reported the effects of baking on phenols and antioxidant capacity of different types of cereal-based products [86,100,101,102,103,104] highlighting the role of cereals, bread formulations, baking parameters, as well as extraction and analytical methods. Our results on breads—decrease in phenolic content and increase in DPPH inhibition—could be attributable to Maillard reaction products such as melanoidins that possess antioxidant properties [105,106] but are also able to bind polyphenols to their protein backbones [107], thus determining a decrease in phenol content. Moreover, our findings can be explained by many factors acting synergistically such as the presence of antioxidant compounds apart from phenols [108], the formation of thermally induced degradation products [109], and polyphenol-polysaccharide complexes [110] that increase the antioxidant scavenging activity. The addition of the multistrain starter mother sourdough positively affected the breads produced, inducing either an increase or a restrained decrease in antioxidant activity and phenolic content. The anticipated health benefits are linked to the consumption of a portion of bread (100 g), which will deliver bioactive (antioxidant) compounds to the human body with the potential to prevent diseases [111]. However, in order to estimate the degree of such health benefits, further studies considering the bioavailability of these compounds are necessary.

Wheat dough texture parameters are important for dough handling properties, dough machinability, and as predictors of bread quality [112]. The texture parameters of the artisanal Calabrian sourdoughs reflect the interesting variability in bread-making; each component of the dough formulation such as type of flour, water, yeast or mother dough, and kneading method are responsible for the final characteristics of the bread [59,113]. The sourdough made in the laboratory (SD) is stickier, less hard, and less extensible compared to the majority of the sourdoughs made at the bakery. Most of the rheological parameters of the novel sourdoughs differentiate them from the traditional ones; this could be explained by considering the low pH due probably to a synergistic effect of traditional sourdough microbiota and the multistrain starter added (PF7 + SD m; PF9 + SD m) [103]. The TPA parameters of the breads produced at the bakery are comparable with data reported by Casado et al. [114]. The addition of the multistrain starter mother sourdough affected the rheological properties of the novel formulated breads. In particular, the PF9 M bread, compared to the PF9 F bread was less hard and was characterized by less chewiness (rubbery texture during mastication), maintaining its springiness (size and shape recovery after compression); features reported for the acceptability of bread [115].

## 5. Conclusions

This study proposed a scientific approach to artisanal sourdough bread-making aiming at improving the characteristics of bread. The sourdough made using the multistrain starter had a phenolic content and antioxidant activity similar to artisanal sourdoughs made with durum wheat studied in this work. This confirms the usefulness of the starter used in the laboratory sourdough formulation. The novel sourdough bread PF7 M had an increase of 170% and 8% for phenolic content and DPPH inhibition capacity, respectively; while the novel sourdough bread PF9 M showed an improvement in textural parameters.

In conclusion, using a multistrain starter in artisanal bread-making can improve the antioxidant and textural properties of breads. Future research could optimize the entire process-type of flour, choice of starter, fermentation time, and baking to improve breads simultaneously both from an antioxidant and sensorial point of view. Moreover, studying the bioavailability of sourdough breads’ bioactive compounds may provide useful knowledge regarding their dietary health benefits.

## Figures and Tables

**Table 1:** pH and total titratable acidity (TTA) of mother doughs and sourdoughs produced at the bakery plant.

Sourdoughs	pH	TTA
SD m	3.74 ± 0.02 ^f^	9.60 ± 0.05 ^a^
PF7	5.55 ± 0.01 ^a^	2.75 ± 0.03 ^e^
PF9	3.90 ± 0.07 ^e^	7.80 ± 0.04 ^b^
PF7 F	5.64 ± 0.07 ^a^	1.55 ± 0.02 ^f^
PF9 F	4.52 ± 0.03 ^b^	4.80 ± 0.05 ^d^
PF7 M	4.19 ± 0.03 ^c^	6.22 ± 0.04 ^c^
PF9 M	4.04 ± 0.02 ^d^	7.89 ± 0.05 ^b^
Statistical significance	***	***

F: bakery traditional sourdough. M: novel sourdough made with the addition of SD to the bakeries’ mother doughs. Mean values in each column with different letters were significantly different according to the Tukey’s test (***, *p* ≤ 0.001).

**Table 2:** Organic acids of sourdoughs and breads produced at laboratory and bakery experiment.

Samples		Organic Acids		QF
		Lactic Acid (mg/g)	Acetic Acid (mg/g)	
**Dough**	SD	4.71 ± 0.09 ^a^	0.99 ± 0.01 ^b^	3.18
	SDC	3.20 ± 0.08 ^b^	2.08 ± 0.05 ^a^	1.02
	PF7 F	0.44 ± 0.10 ^e^	0.46 ± 0.01 ^c^	0.64
	PF7 M	1.96 ± 0.04 ^d^	0.35 ± 0.00 ^d^	3.77
	PF9 F	3.25 ± 0.10 ^b^	0.46 ± 0.02 ^c^	4.70
	PF9 M	2.28 ± 0.06 ^c^	0.34 ± 0.03 ^d^	4.50
**Bread**	SD	3.78 ± 0.04 ^A^	1.75 ± 0.09 ^B^	-
	SDC	3.15 ± 0.06 ^B^	1.99 ± 0.11 ^A^	-
	PF7 F	0.35 ± 0.02 ^F^	0.27 ± 0.02 ^C^	-
	PF7 M	0.66 ± 0.02 ^E^	0.20 ± 0.02 ^C^	-
	PF9 F	1.02 ± 0.07 ^D^	0.32 ± 0.02 ^C^	-
	PF9 M	1.19 ± 0.03 ^C^	0.22 ± 0.03 ^C^	-
Statistical significance		***	***	

F: bakery traditional sourdough. M: novel sourdough made with the addition of SD to the bakeries’ mother doughs. Mean values in a vertical column with different letters were significantly different according to Tukey’s test (***, *p* ≤ 0.001). Sourdoughs and breads were analyzed separately and distinguished with small and capital letters, respectively.

**Table 3:** DPPH inhibition (%) and total phenolic content of the sourdoughs and breads made in the laboratory.

Samples		DPPH ^a^				Total Phenols ^b^			
		0 h	8 h	21 h	2 months	0 h	8 h	21 h	2 months
**Sourdoughs**	SD	13.09 ± 0.07 ^c^	17.28 ± 0.08 ^b^	20.07 ± 0.16 ^a^	12.46 ± 0.22 ^d^	120.51 ± 0.54 ^e^	128.64 ± 0.47 ^d^	168.96 ± 0.24 ^c^	178.58 ± 0.19 ^b^
	SDC	10.38 ± 0.18 ^e^	9.65 ± 0.03 ^f^	12.28 ± 0.17 ^d^	10.50 ± 0.19 ^e^	100.90 ± 0.23 ^h^	102.75 ± 0.21 ^g^	103.86 ± 0.13 ^f^	183.02 ± 0.25 ^a^
	Statistical significance	***				***			
**Breads**	SD	26.55 ± 0.23 ^A^				80.56 ± 0.00 ^A^			
	SDC	24.69 ± 0.24 ^B^				72.79 ± 0.00 ^B^			
	Statistical significance	***				***			

^a^ % of inhibition. ^b^ mg of gallic acid/100g of sourdough and bread. Statistical analysis was carried out separately for the two parameters. Mean values with different letters were significantly different according to Tukey’s test (***, *p* ≤ 0.001). Sourdoughs and breads were analyzed separately. Means of sourdoughs were horizontally distinguished with small letters. Means of breads were vertically distinguished with capital letters.

**Table 4:** DPPH inhibition (%) and total phenolic content of the sourdoughs and breads produced at the bakery.

Analyses	Sourdoughs				Breads			
	PF7 F	PF9 F	PF7 M	PF9 M	PF7 F	PF9 F	PF7 M	PF9 M
DPPH ^a^	12.49 ± 0.12 ^c^	17.68 ± 0.24 ^a^	13.53 ± 0.26 ^b^	11.71 ± 0.20 ^d^	31.98 ± 0.14 ^B^	30.91 ± 0.25 ^C^	32.69 ± 0.27 ^A^	24.04 ± 0.36 ^D^
Statistical significance	***				***			
Total phenols ^b^	19.53 ± 0.04 ^d^	30.63 ± 0.34 ^b^	52.82 ± 0.17 ^a^	26.19 ± 0.08 ^c^	18.05 ± 0.11 ^B^	15.09 ± 0.09 ^C^	25.82 ± 0.13 ^A^	11.76 ± 0.10 ^D^
Statistical significance	***				***			

^a^ % of inhibition. ^b^ mg of gallic acid/100 g of sourdough and bread. F: bakery traditional sourdough/bread. M: novel sourdough/bread made with the addition of SD to the bakeries’ mother doughs. Values in the line with different letters were significantly different according to the Tukey’s test (***, *p* ≤ 0.001). Sourdoughs and breads were analyzed separately and distinguished with small and capital letters, respectively.

**Table 5:** Rheological characteristics (**a**) Stickiness and Hardness and (**b**) Firmness, Adhesiveness, and Extensibility of the sourdoughs made in the laboratory. Superscript letters refer to the statistical analysis.

**Sourdough**	**Stickiness Test**				**Penetration Test**
	**Stickiness (g)**	**Work of Adhesion (g·s)**		**Dough Strength/Cohesiveness (mm)**	**Hardness (g)**
SD	111.82 ± 3.92 ^a^	19.22 ± 0.50 ^a^		6.37 ± 0.04 ^a^	46.37 ± 3.87 ^a^
SDC	114.40 ± 2.80 ^a^	16.82 ± 1.21 ^a^		6.31 ± 0.08 ^a^	42.06 ± 2.71 ^a^
**(a)**					
**Sourdough**	**Warburtons Test**			**Kieffer Test**	
	**Firmness (kg)**	**Work of Compression (kg·s)**	**Adhesiveness Peak (kg)**	**Resistance to Extensibility (g)**	**Extensibility (mm)**
SD	0.59 ± 0.14 ^a^	4.19 ± 0.62 ^a^	0.75 ± 0.14 ^a^	6.48 ± 0.43 ^a^	0.23 ± 0.30 ^a^
SDC	0.47 ± 0.06 ^a^	3.33 ± 0.56 ^a^	0.60 ± 0.05 ^a^	5.22 ± 0.39 ^b^	0.19 ± 0.28 ^a^
**(b)**					

**Table 6:** Rheological characteristics (**a**) Stickiness and Hardness and (**b**) Firmness, Adhesiveness, and Extensibility of the sourdoughs made at the bakery. Superscript letters refer to the statistical analysis.

**Sourdough**	**Stickiness Test**			**Penetration Test**	
	**Stickiness (g)**	**Work of Adhesion (g·s)**	**Dough Strength/Cohesiveness (mm)**	**Hardness (g)**	
Mother sourdough (PF7 + SD m)	37.98 ± 1.87 ^b^	3.43 ± 0.54 ^a^	1.62 ± 0.33 ^a^	150.23 ± 26.25 ^a^	
Mother sourdough (PF9 + SD m)	79.58 ± 13.46 ^ab^	4.33 ± 1.44 ^a^	1.24 ± 0.48 ^a^	133.65 ± 14.44 ^ab^	
PF7 F	72.70 ± 10.64 ^ab^	5.94 ± 2.20 ^a^	1.87 ± 0.51 ^a^	62.73 ± 15.67 ^b^	
PF9 F	99.52 ± 14.26 ^a^	7.50 ± 1.85 ^a^	1.87 ± 0.39 ^a^	88.46 ± 14.86 ^ab^	
PF7 M	76.29 ± 16.82 ^ab^	5.31 ± 2.01 ^a^	1.70 ± 0.56 ^a^	96.00 ± 13.46 ^ab^	
PF9 M	105.49 ± 11.12 ^a^	9.22 ± 2.32 ^a^	2.34 ± 0.66 ^a^	75.79 ± 22.03 ^ab^	
**(a)**					
**Sourdough**	**Warburtons Test**			**Kieffer Test**	
	**Firmness (kg)**	**Work of Compression (kg·s)**	**Adhesiveness Peak (kg)**	**Resistance to Extensibility (g)**	**Extensibility (mm)**
Mother sourdough (PF7 + SDm)	3.38 ± 0.38 ^a^	19.04 ± 4.99 ^a^	−0.73 ± 0.11 ^a^	33.96 ± 7.49 ^a^	35.94 ± 1.72 ^ab^
Mother sourdough (PF9 + SDm)	1.38 ± 0.23 ^a^	11.13 ± 1.79 ^a^	−1.62 ± 0.28 ^a^	7.94 ± 1.96 ^a^	24.70 ± 3.84 ^b^
PF7 F	2.98 ± 1.30 ^a^	10.86 ± 3.80 ^a^	−1.08 ± 0.31 ^a^	39.95 ± 8.44 ^a^	40.06 ± 4.07 ^a^
PF9 F	3.34 ± 0.85 ^a^	14.16 ± 3.26 ^a^	−1.13 ± 0.15 ^a^	28.49 ± 11.64 ^a^	37.73 ± 2.26 ^a^
PF7 M	4.38 ± 0.89 ^a^	16.46 ± 3.55 ^a^	−0.92 ± 0.10 ^a^	30.20 ± 8.23 ^a^	36.23 ± 1.68 ^ab^
PF9 M	2.13 ± 1.56 ^a^	10.94 ± 8.65 ^a^	−0.92 ± 0.65 ^a^	24.84 ± 5.89 ^a^	36.68 ± 3.45 ^ab^
**(b)**					

## Supplementary Materials

The following are available online at https://www.mdpi.com/2304-8158/9/9/1258/s1, Table S1: DPPH and total phenolic compounds of ten traditional Calabrian sourdoughs and dough produced with baker’s yeast; Table S2: Rheological attributes (a) Stickiness and Hardness and (b) Firmness, Adhesiveness, and Extensibility of ten traditional Calabrian sourdoughs and dough produced with baker’s yeast.
