# Supplementary material for: Persistence and Effect of a Multistrain Starter Culture on Antioxidant and Rheological Properties of Novel Wheat Sourdoughs and Bread
Source: Foods. 2020 Sep 8;9(9):1258. doi: 10.3390/foods9091258 (PMC7555968; doi:10.3390/foods9091258)
Supplement: Supplementary file 1 [file foods-09-01258-s001.pdf]

Table S1. DPPH and total phenolic compounds of ten traditional Calabrian sourdoughs and dough produced with baker's yeast.

**Doughs**	**DPPH**^{a}	**Total phenols**^{b}
PF1	20.38±0.12^{e}	173.41±0.16^{e}
PF2	19.27±0.14^{f}	97.64±0.05^{i}
PF3	14.54±0.18^{h}	178.87±0.12^{d}
PF4	21.17±0.19^{d}	183.66±0.11^{b}
PF5	22.27±0.23^{c}	95.83±0.06^{l}
PF6	29.60±0.17^{b}	108.38±0.09^{h}
PF7	21.62±0.12^{d}	110.35±0.22^{g}
PF8	20.44±0.26^{e}	179.35±0.32^{c}
PF9	14.14±0.19^{h}	148.92±0.05^{f}
PF10	35.82±0.25^{a}	195.62±0.04^{a}
PFC	17.77±0.23^{g}	71.61±0.10^{m}
Statistical significance	***	***

^{a}% of inhibition

^{b}mg of gallic acid/100 g of dough

Means values in a vertical column with different letters were significantly different according to the Tukey's test (***, $p \leq 0.001$).

Table S2. Rheological attributes (**a**) Stickiness and Hardness and (**b**) Firmness, Adhesiveness, extensibility of ten traditional Calabrian sourdoughs and dough produced with baker's yeast.

Dough	Stickiness test			Penetration test
	Stickiness (g)	Work of Adhesion (g.sec)	Dough strength/ Cohesiveness (mm)	Hardness (g)
PF1	221.16±53.49[ab]	8.82±1.62[ab]	1.26±0.20[b]	22.32±3.58[d]
PF2	142.25±11.37[bc]	16.36±1.09[a]	4.50±0.11[a]	67.39±13.26[cd]
PF3	48.38±15.67[cd]	4.74±2.24[b]	2.01±0.87[ab]	103.18±16.46[bd]
PF4	261.76±32.18[a]	15.04±2.75[a]	3.76±0.31[ab]	17.59±5.63[d]
PF5	43.07±7.80[d]	2.28±0.71[b]	1.04±0.23[b]	177.56±24.86[abc]
PF6	71.95±7.41[cd]	9.35±1.14[ab]	3.58±0.45[ab]	85.58±10.14[bd]
PF7	47.41±6.27[cd]	2.68±0.87[b]	1.02±0.27[b]	217.74±29.65[ab]
PF8	40.91±7.12[d]	2.12±0.87[b]	0.95±0.36[b]	281.76±75.14[a]
PF9	41.69±9.98[d]	2.69±1.22[b]	1.21±0.47[b]	197.36±27.43[abc]
PF10	79.19±8.41[cd]	10.14±4.35[ab]	3.79±1.54[ab]	119.26±13.95[bd]
PFC	78.89±16.16[cd]	10.24±1.98[ab]	3.71±0.57[ab]	33.66±3.53[d]

(a)

Dough	Warburtons test			Kieffer test	
	Firmness (kg)	Work of Compression (kg.sec)	Adhesiveness peak (kg)	Resistance to extensibility (g)	Extensibility (mm)
PF1	0.28±0.08[c]	1.45±0.64[c]	- 0.25±0.02[a]	nd*	nd*
PF2	nd*	nd*	nd*	24.03±6.57[ab]	- 15.26±2.84[cd]
PF3	0.72±0.20[c]	4.85±1.57[c]	- 1.49±0.28[a]	12.96±1.10[b]	11.00±4.13[ab]
PF4	nd*	nd*	nd*	nd*	nd*
PF5	2.93±0.42[ac]	17.92±3.05[ac]	- 1.59±0.70[a]	43.68±2.56[a]	- 16.34±2.08[d]
PF6	nd*	nd*	nd*	30.25±6.43[ab]	15.08±2.96[ab]
PF7	8.62±2.42[a]	52.09±16.31[a]	- 1.99±0.79[a]	32.85±7.43[ab]	12.56±1.50[ab]
PF8	7.04±1.78[ac]	41.44±5.83[ac]	- 1.38±0.19[a]	32.54±8.07[ab]	11.54±1.12[ab]
PF9	7.66±2.91[ab]	47.82±17.19[ab]	- 1.7±0.48[a]	44.84±6.58[a]	16.76±4.23[a]
PF10	1.28±0.31[bc]	7.98±1.82[bc]	- 1.43±0.26[a]	12.93±4.73[b]	- 0.02±0.00[bc]
PFC	0.65±0.43[c]	2.70±1.02[c]	- 0.28±0.06[a]	13.91±2.67[b]	- 12.20±7.53[cd]

*nd: not determined

(b)
